# Mammography-Based Radiomics for Prediction of Nodal Status and Disease Burden in Breast Cancer: A Temporally Validated Study

**DOI:** 10.3390/cancers18162566

**Published:** 2026-08-10

**Authors:** Grzegorz Chmielewski, Rafał Stando, Hubert S. Gabryś, Maksym Fritsak, Stephanie Tanadini-Lang, Matthias Guckenberger, Stanisław Góźdź

**Affiliations:** 1 Department of Radiation Oncology, Holy Cross Cancer Center, 25-734 Kielce, Poland; rafal.stando@onkol.kielce.pl (R.S.); 2 Collegium Medicum, Jan Kochanowski University, 25-317 Kielce, Poland; stanislaw.gozdz@onkol.kielce.pl; 3 Department of Radiation Oncology, University Hospital Zurich, University of Zurich, 8091 Zurich, Switzerland; hubert.gabrys@usz.ch (H.S.G.); maksym.fritsak@usz.ch (M.F.); stephanie.tanadini-lang@usz.ch (S.T.-L.); matthias.guckenberger@usz.ch (M.G.); 4 Department of Clinical Oncology, Holy Cross Cancer Center, 25-734 Kielce, Poland

**Keywords:** breast cancer, radiomics, mammography, mammography-based radiomics

## Abstract

**Objectives:** We evaluated the performance of mammography-based radiomics in prediction of clinical nodal status, clinical tumor stage, clinical disease stage, status of the PIK3CA mutation and concordance with the radiologist-assessed BI-RADS category. Mammography is often the first imaging modality performed in the screening or diagnostic workup of breast cancer. **Materials and Methods:** In this single-center retrospective study, we included 102 histopathologically confirmed cases of breast cancer from 100 patients. The tumor region and whole-breast parenchyma were contoured on 368 craniocaudal and mediolateral-oblique mammograms. Cases were temporally split into training and test sets by histopathological diagnosis date (training: 70%, held-out test: 30%). Six linear models were tuned by 5-fold x 3-repeat stratified cross-validation. The winning model per endpoint was applied once to the test set. We report AUCs with 95% confidence intervals, permutation *p*-values and Benjamini–Hochberg false discovery rate (BH-FDR) correction across five endpoints. **Results:** Three of the studied endpoints reached significance after BH-FDR correction: clinical nodal status (cN0 vs. cN-positive; AUC: 0.726 (95% CI: 0.501–0.886)), clinical tumor stage (cT1-2 vs. cT3-4; AUC: 0.792 (95% CI: 0.575–0.929)) and overall disease stage (I–II vs. III–IV; AUC: 0.778 (95% CI: 0.572–0.917)). Mammography-based radiomics failed to predict the presence of PIK3CA mutation and concordance with radiologist-assessed BI-RADS category. **Conclusions:** Mammography-based radiomics has shown a hypothesis-generating discriminative value for differentiation between cN-negative and cN-positive disease, early from advanced clinical tumor stage, and early from advanced overall disease stage in breast cancer. Mammography-based radiomics did not predict PIK3CA status and did not discriminate between radiologist-assessed BI-RADS 4 and 5 groups.

## Simple Summary

We assess whether the most widespread breast imaging modality carries information beyond the basic differentiation between benign and malignant lesions. Mammography-based radiomic models distinguished cN-negative from cN-positive disease, early from advanced clinical tumor stage, and early from advanced overall stage; PIK3CA and BI-RADS predictions failed. In this study, a preliminary, hypothesis-generating radiomic signal was found for prediction of nodal involvement and advanced disease. External validation in larger, multicenter cohorts is required before any use for risk stratification can be considered.

## 1. Introduction

Breast cancer (BC) is the most common cancer in women (excluding non-melanoma skin cancer), with 361,859 new cases and 92,749 deaths in the European Union in 2024 [1]. Mammography (MMG) is the most widely used breast imaging modality worldwide. In many countries, it is part of screening programs aimed at early detection of BC. In the United States, approximately 39 to 40 million MMG procedures are performed annually [2]. Given its reported sensitivity of approximately 72%, relatively low cost, and high availability, it remains the cornerstone of BC screening [3].

Radiomics focuses on extracting quantitative features from medical images. It is gaining momentum in oncology, as the field continually seeks novel and more robust predictive methods. The main promise of radiomics is that it captures what is imperceptible to the human eye, such as subtle variations in shape, texture, and signal intensity [4]. These features, when combined with clinical, histopathological, and genetic variables, can be used to develop predictive models.

Radiomics has been investigated in BC since the field’s inception. Initial reports appeared in 2015, followed by numerous studies [5]. Radiomics and multi-omics approaches have been successful in predicting multiple characteristics of BC, including hormone receptor and HER2 status, response to treatment, and risk of recurrence, among others [6,7,8]. However, the majority of BC radiomics and multi-omics studies use magnetic resonance imaging (MRI)-derived features. While MRI offers superior soft tissue contrast and three-dimensional evaluation of the breast, it is not routinely used as the primary diagnostic imaging modality in the general population.

In this study, we aimed to evaluate whether MMG-based radiomics, leveraging a more widely available modality, can predict the disease burden, concordance with radiologist-assessed BI-RADS category and the status of PIK3CA mutation in BC.

## 2. Materials and Methods

### 2.1. Patient Cohort

**Figure 1:**
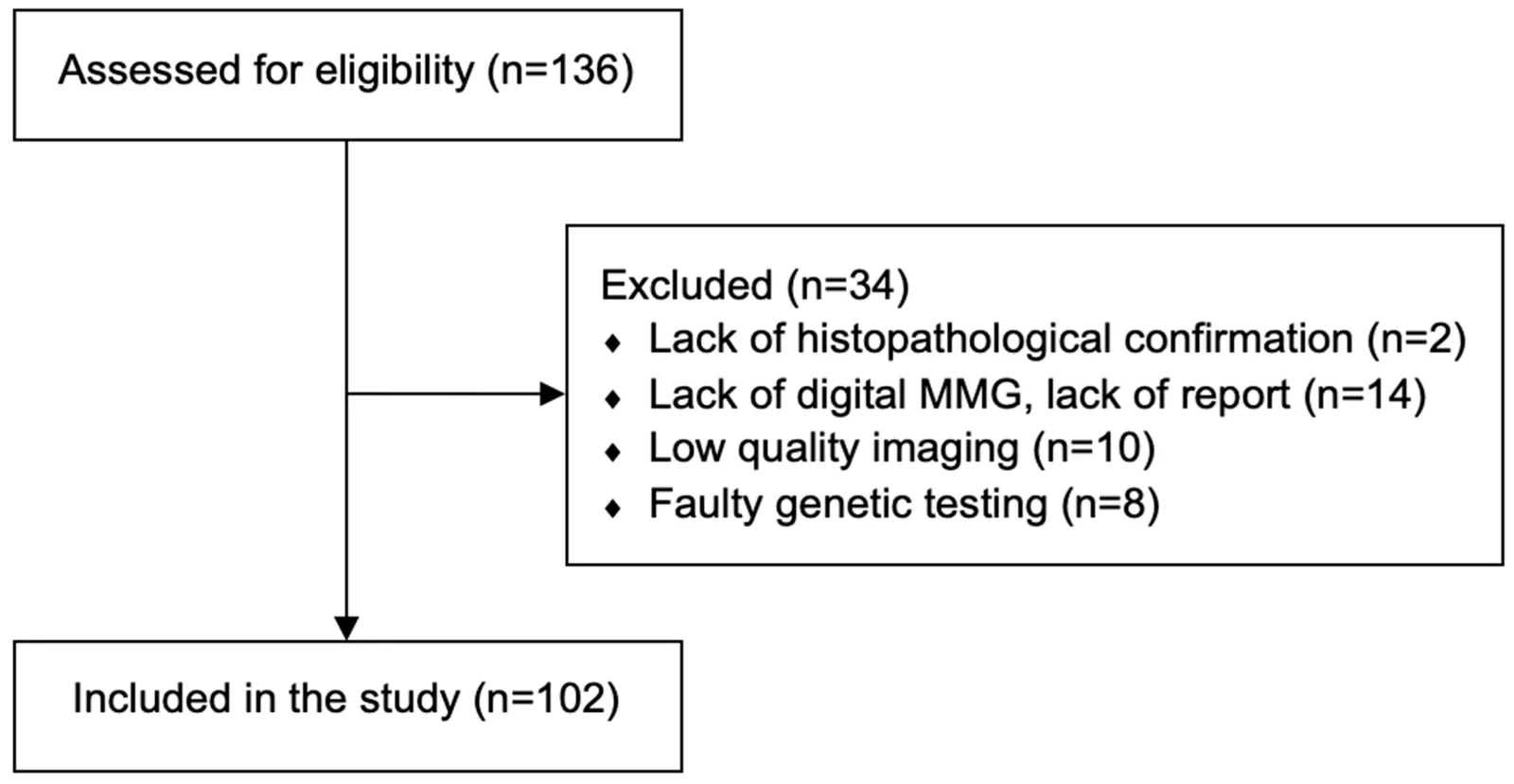
CONSORT-style case flow diagram for inclusion in the study. A total of 136 breast cancer cases from 134 female patients were screened from the institutional archive. The exclusion criteria included lack of histopathological confirmation of breast cancer, lack of digital mammogram in the hospital’s archives, lack of written radiologist’s report, insufficient quality of imaging and faulty genetic testing. A total of 102 cases from 100 patients were eventually included in the study.

In this single-center retrospective study, we screened 136 BC cases from 134 female patients treated at Holy Cross Cancer Center, Kielce, Poland. Due to the retrospective nature of the study, with all data collected during the diagnostic and treatment process, institutional review board approval was not required. The inclusion criteria were histopathological confirmation of BC and the presence of a pre-treatment digital mammogram in the hospital’s archives. Exclusion criteria were lack of histopathological confirmation, lack of mammograms, unavailable radiologist’s report for the MMG, low quality of imaging or faulty genetic testing due to sample degradation. After the initial screening, 34 cases from 34 patients were excluded from the study. Utilized methods are reported as per TRIPOD+AI guidelines [9]. Figure 1 demonstrates the inclusion process.

### 2.2. Endpoints

**Figure 2:**
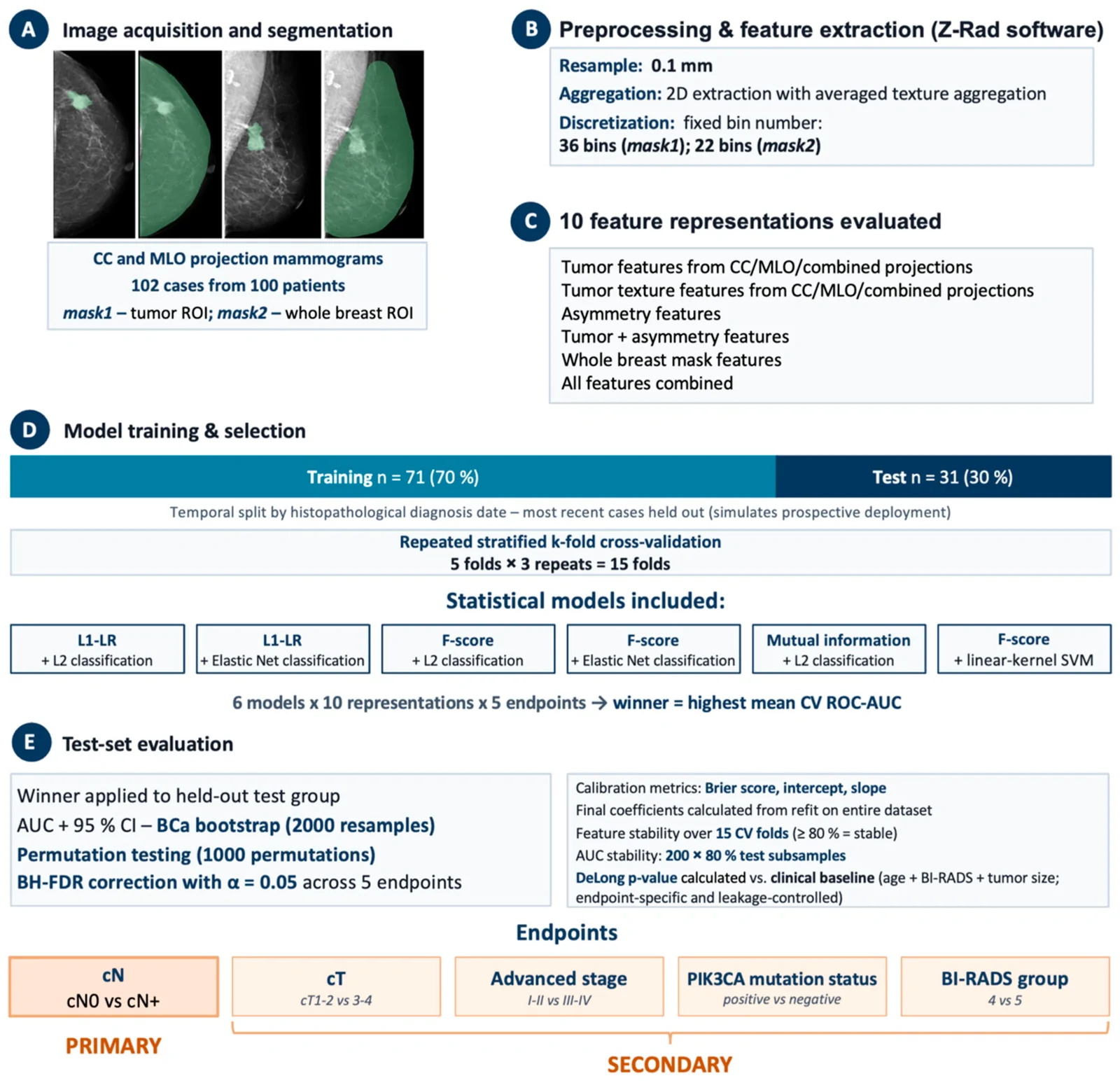
Methodological workflow of the study. Abbreviations: CC, craniocaudal; MLO, mediolateral oblique; L1-LR, L1-regularized regression; ANOVA, Analysis of Variance; ROC-AUC, area under the receiver operating characteristic curve; CI, confidence interval; BCa, Bias-Corrected and Accelerated; BH-FDR, Benjamini–Hochberg false discovery rate; cN, clinical nodal status; cT, clinical tumor status; BI-RADS, Breast Imaging Reporting and Data System.

The primary endpoint was the evaluation of prognostic performance of MMG-derived radiomic features for prediction of the presence of imaging-defined nodal status in the pre-surgical diagnostic process (cN status, assigned from the pre-treatment ultrasound as per American Joint Committee on Cancer (AJCC) guidelines). Secondary endpoints were the radiomics-based prediction of clinical tumor stage (cT), overall clinical disease stage, presence of PIK3CA mutation, and concordance with radiologist-assessed BI-RADS 4 and 5 groups. The methodological workflow of this study is summarized in Figure 2.

### 2.3. Image Acquisition

Mammograms were taken using the following MMG hardware: Siemens Mammomat Inspiration, with kilovoltage peaks up to 35 kV, a tungsten anode and a 0.05 mm rhodium filter, and Hologic Selenia Dimensions and Lorad Selenia systems with kilovoltage peaks up to 33 kV, molybdenum anodes and 0.03 mm rhodium filters. In total, 57.6% of the mammograms were performed using the Siemens system, and the remaining 42.4% were taken using the Hologic systems, based on the retrieved acquisition metadata. Scanner-related variability was examined by comparing each selected feature between acquisition vendors (utilizing Mann–Whitney U test). Open source, Image Biomarker Standardisation Initiative (IBSI)-compliant Z-Rad software, version 25.4 (University Hospital Zurich, Zurich, Switzerland) was used for image and mask preprocessing and radiomic features extraction [10]. All the images and masks were preprocessed, with a resample resolution of 0.1 mm, linear image interpolation and nearest-neighbor mask interpolation. The radiomic features were extracted from NIfTI files, with 2D averaged texture aggregation and fixed bin number discretization—36 bins for mask1 and 22 for mask2 (based on IQRs of pooled tumor and breast tissue intensities in the training subset, applied unchanged to test). A total of 138 radiomic features were extracted for every segmentation. The full list of extracted radiomic features is provided in the Supplementary Table S1.

#### 2.3.1. Image Segmentation

Mammograms of ‘tumor-side’ and ‘healthy’ breasts in craniocaudal (CC) and mediolateral oblique (MLO) projections were collected. Segmentations were made for the tumor region (mask1) and for the whole breast, excluding skin and nipple (mask2) for the ‘tumor-side’ breast, and a whole-breast segmentation for the ‘healthy’ breast. The segmentations were performed using 3D Slicer software, version 5.8.0 and saved as a binary labelmap in NIfTI format for later processing [11]. All segmentations were performed manually. Segmentation of the tumor region was performed according to a written radiologist’s report in all cases.

An assessment of segmentation reproducibility was performed on a prespecified, 30-MMG subsample (15 CC, 15 MLO mammograms) from 30 patients. The 30 mammograms were randomly selected from the tumor-side mammograms. A second reader independently contoured the tumor region and the whole breast on these mammograms, based on the same written radiologist’s report, blinded to the initial contours. The features were re-extracted utilizing the identical Z-Rad pipeline. Inter-observer agreement was assessed as the intraclass correlation coefficient (ICC (2,1)) with 95% confidence intervals from a nonparametric bootstrap over cases, with 2000 resamples. Features with an ICC of at least 0.75 were considered reproducible. The Dice similarity coefficient was calculated between the segmentations. The models were not refitted based on the results of this analysis.

#### 2.3.2. Data Aggregation

Radiomic features were extracted per segmentation and aggregated to one row per case. Each case therefore carries four feature blocks per view (CC, MLO): tumor ROI (mask1) when present, ipsilateral whole breast (mask2), contralateral whole breast (mask2), and the ipsilateral-minus-contralateral difference of the whole-breast features. Patients with bilateral disease contributed one case per affected breast, giving 102 cases from 100 patients. Clinical variables recorded across multiple rows per patient were collapsed by a worst-case rule: the maximum for continuous variables, and a positive value if any record was positive for binary endpoints.

#### 2.3.3. Statistical Analysis

For model validation, cases were divided into training (70%) and test (30%) sets with a temporal split based on the histopathological diagnosis date, with the most recent cases in the test set. This approach simulates a prospective deployment. The split was fixed throughout analyses. The random seed was fixed at 42 for reproducibility.

We evaluated 10 feature subsets to assess the extent to which they contribute to the predictive performance of MMG-based radiomics. The subsets included: tumor ROI-based features (all features and exclusively texture features, separately for CC, MLO views and combined), contralateral asymmetry features, a combination of tumor features and asymmetry features, and whole-breast mask features, as well as all features combined. For each of the described representations, we trained 6 linear models. The linear models included: L1-regularized logistic regression as selector with L2-regularized logistic regression or elastic net classifier, an F-score univariate filter with L2-regularized logistic regression or an elastic net classifier, a mutual information univariate filter with an L2-regularized logistic regression classifier, and an F-score univariate filter with a linear-kernel calibrated support vector machine classifier. The non-linear models (random forest and gradient boosting) were excluded during the initial analyses due to severe overfitting at the available sample size. For all the models, we employed median imputation for missing values, variance thresholding, feature standardization with the Z-score, and feature selection (limited to k = 2–5 features). The data missingness report is available in the Supplementary Table S2. Hyperparameter tuning was performed using a grid search within repeated stratified k-fold cross-validation (CV; 5 folds × 3 repeats) on the training set. The winning model was the model with the highest mean CV area under the receiver operating characteristic curve (ROC AUC) for each endpoint. Both tumors from the same patient could appear in the same CV fold. Given the negligible number of bilateral cases (2/100), no patient-level grouping was applied.

The winning model was applied to the temporally split test set. The achieved discrimination was assessed using the AUC. Confidence intervals (CIs) were calculated using bias-corrected, accelerated bootstrapping with 2000 resamples. Statistical significance was calculated using permutation testing (1000 permutations, yielding a minimum *p*-value of 0.001), and endpoints with permutation *p*-values of less than 0.05 were considered statistically significant. Benjamini–Hochberg correction with a false discovery rate of 0.05 was applied across the five endpoints [12]. The winning model with its optimal hyperparameters was refitted on the entire dataset to obtain the final set of features and their coefficients.

In the paper, we report the generalization performance of the test set. We also assessed the calibration of the model by calculating the Brier score, intercept and slope. After that step, the model was compared to a clinical baseline model (consisting of age at diagnosis, tumor size and BI-RADS group). The clinical model was trained using L2-regularized logistic regression. The comparison was performed using the DeLong test for paired AUCs. BI-RADS group was excluded from the analysis for BI-RADS endpoint to prevent leakage. To address selection variability, the winning pipeline was refitted on each of the 15 CV folds for cN, cT and advanced-stage endpoints, and feature selection frequency was reported. Features selected in ≥80% of folds are considered stable. To assess the stability of test set performance, the evaluation was repeated 200 times on random 80% subsamples of the test set.

Model optimism was assessed using bootstrap internal validation (development-set resampling), reporting optimism-corrected AUCs. Calibration was displayed with reliability curves and bootstrap 95% confidence intervals for the Brier score, calibration intercept and slope. Clinical net benefit was assessed by decision curve analysis across threshold probabilities of 0.05–0.50. A PROBAST-AI-style risk-of-bias self-assessment table is presented in the Supplementary Table S3.

All analyses were performed in Python 3.13 with scikit-learn 1.8, statsmodels, SciPy, and matplotlib libraries [13,14,15,16]. Claude Code (artificial intelligence agent; Anthropic, San Francisco, CA, USA) was used for code debugging.

## 3. Results

### 3.1. Cohort Baseline Characteristics and Sample Size

After applying the inclusion and exclusion criteria, 102 cases from 100 patients were included in the analysis. The median patient age in the studied group was 63 years (interquartile range, IQR: 49–70 years), while the median age at diagnosis was 59 years (IQR: 47–67 years). In total, 42% of cases were positive for PIK3CA mutation, 49% had clinically suspicious lymph nodes assessed using ultrasound (cN positivity), 34.4% were classified as BI-RADS 4 and 65.6% as BI-RADS 5 (BI-RADS 6 was excluded from analyses as it stands for pathologically confirmed cancer), 58.4% were stage I or II, and 41.6% were stage III or IV at diagnosis. In total, 58.8% were clinically staged as cT1 or cT2, while 40.2% were staged as cT3 or cT4. Baseline characteristics of the cohort, including the remaining hormonal, molecular and clinical features, with the split into training and test subsets are presented in Table 1.

We calculated the events-per-variable (EPV) index using the smaller class as the event count. For all three positive endpoints, EPV fell below the TRIPOD-recommended 10, potentially limiting the generalizability of the results. This issue is further addressed in the limitations section.

### 3.2. Primary Endpoint: Prediction of cN Status

The primary results of the analysis are presented in Table 2. MMG-based radiomics showed statistically significant discrimination for clinical nodal status, clinical tumor stage and overall disease stage in this cohort. The model failed to discriminate BI-RADS 4 vs. 5 groups and the presence of PIK3CA mutation in the studied group. For the primary endpoint, clinical nodal status prediction (cN0 vs. cN1+), the best feature subset was texture features from the MLO projection for the tumor ROI, and the best statistical model was model-based feature selection with L1-regularized logistic regression followed by classification with L2-regularized logistic regression. The CV AUC was 0.684, with the test AUC being 0.726 (95% CI: 0.50–0.89), with a wide confidence interval reflective of a small sample. Statistical significance was confirmed by permutation testing, with a *p*-value of 0.01 and a Benjamini–Hochberg-adjusted q-value of 0.017. Features selected for prediction of cN status were: the GLCM Information Correlation 1 (cm_info_corr1, β = −0.052), the GLDZM gray level variance (dzm_gl_var, β = +0.049), the GLDZM zone distance entropy (dzm_zd_entr, β = +0.137), and the NGLDM low-dependence low-gray-level emphasis (ngl_ldlge, β = −0.103). Given the low EPV and the small test set, we also report bootstrap optimism-corrected AUCs in Table 3. The temporally held-out test AUCs equaled or exceeded these values, indicating no inflation of test performance relative to internal expectation. Repeating the test set evaluation on 200 random 80% subsamples returned median AUCs close to the full test set values (cN status: 0.721, IQR: 0.696–0.757; cT status: 0.792, IQR: 0.755–0.827; clinical stage: 0.777, IQR: 0.743–0.804). This serves as evidence that the reported discrimination was not driven by a small number of individual test cases.

### 3.3. Prediction of cT Status and Overall Disease Stage

We observed stronger results for two of the secondary endpoints: the differentiation of early vs. advanced clinical tumor status (cT1–2 vs. 3–4) and clinical stage (I–II vs. III–IV). For the cT status, the CV AUC was 0.824 and the temporally split test set AUC was 0.792 (95% CI: 0.58–0.93). Similarly, for the advanced-stage prediction, the CV AUC was 0.763 and the test set AUC was 0.778 (CI: 0.57–0.92). Both endpoints were stable through the BH-FDR correction. Radiomic features selected for the positive endpoints are presented together with their coefficients and prevalence across folds in Table 4. Precision–recall curves for all the positive endpoints are provided in the Supplementary Figure S1. Interestingly, for clinical stage prediction, the refit features were not consistently selected during CV (<13%), but intensity-based energy (stat_energy) was selected in 14/15 folds. This likely results from L1 feature selection instability. A stable imaging biomarker identification would require a larger cohort.

### 3.4. Prediction of BI-RADS Group Concordance and PIK3CA Mutation

**Figure 3:**
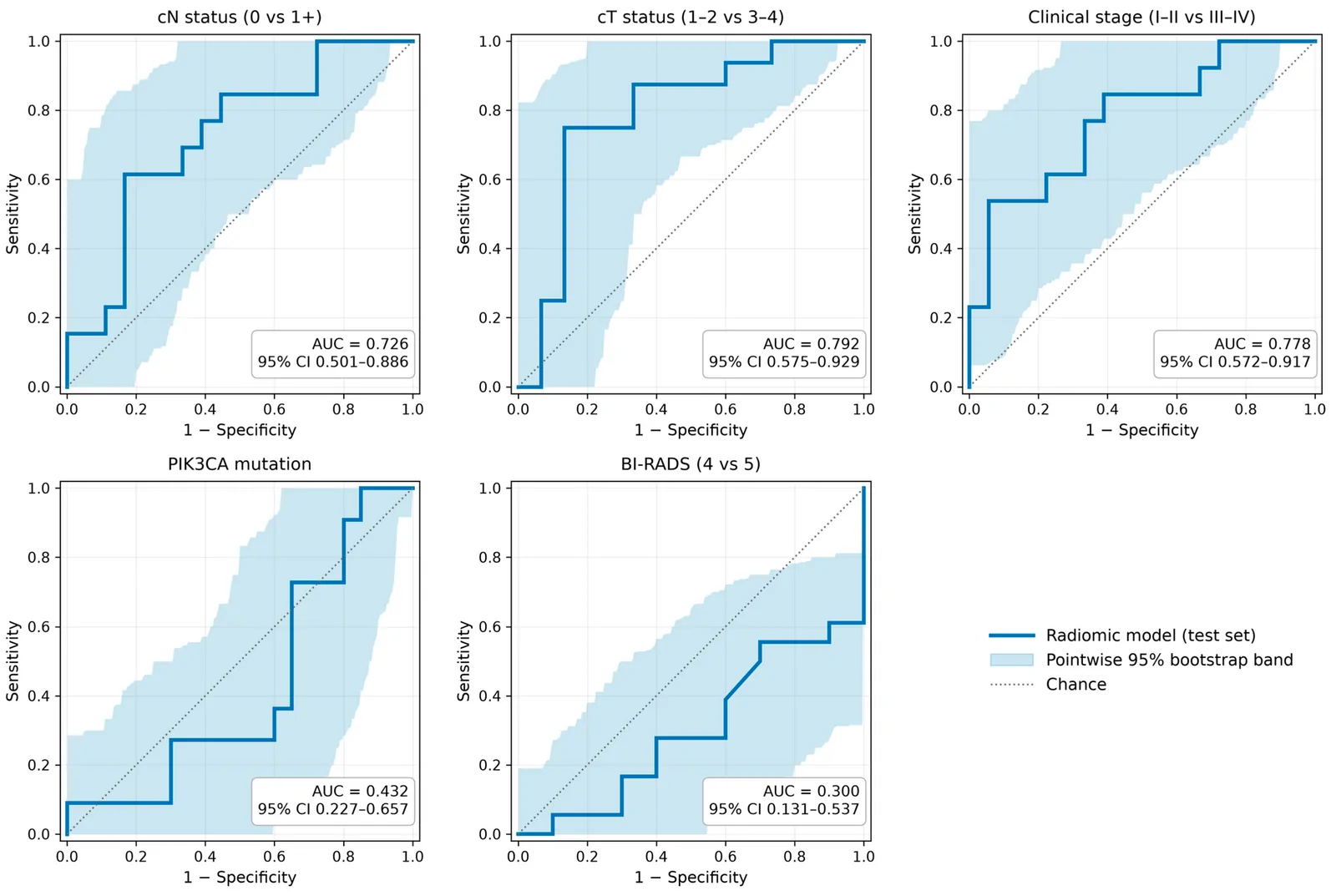
ROC curves for the temporally held-out test set for all five studied endpoints with the AUC values and 95% confidence intervals. The positive endpoints include prediction of clinical nodal status (cN0 vs. cN-positive), clinical tumor status (cT1–2 vs. 3–4) and overall clinical disease stage (stage I–II vs. III–IV). Negative endpoints include prediction of the *PIK3CA* mutation and differentiation between radiologist-assessed BI-RADS groups 4 and 5. Abbreviations: AUC ROC, area under the receiver operating characteristic curve; cN, clinical nodal status; cT, clinical tumor status; BI-RADS, Breast Imaging Reporting and Data System.

The remaining two of the secondary endpoints were clearly negative: differentiation between BI-RADS 4 and 5 groups (test AUC: 0.300) and prediction of PIK3CA status (test AUC: 0.432). A detailed table of calibration metrics for all models is provided in Table 5. ROC curves for all the endpoints are shown in Figure 3.

### 3.5. Comparison with the Clinical Baseline

**Figure 4:**
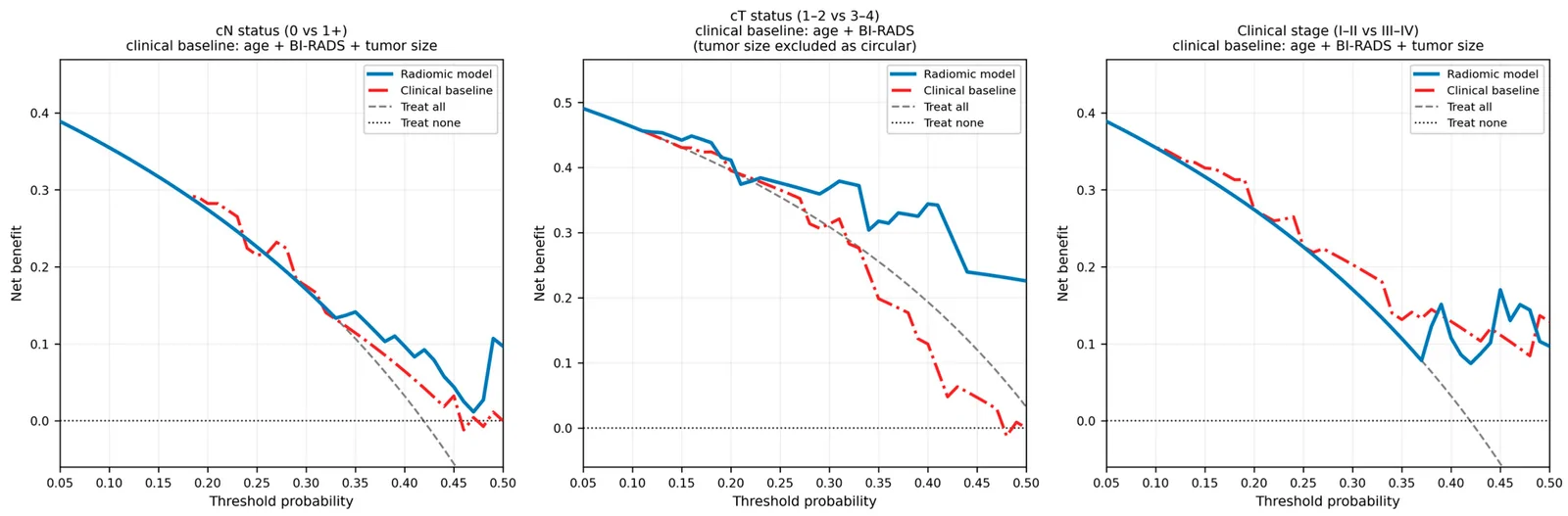
Decision curves for the three positive endpoints in the temporally held-out test set. Net benefit is plotted against threshold probability over 0.05–0.50 for the radiomic model, the clinical baseline and the two strategies (treat all, treat none).

The results were compared to a clinical model, which included three quantifiable features (age + BI-RADS + tumor size from pathology report). For cT status prediction, the clinical model included only age and BI-RADS category. The radiomic model outperformed the clinical baseline in all three positive endpoints (Table 6); however, the differences were not significant. The study was not powered to detect AUC differences between the radiomic models and a clinical baseline at the magnitudes observed. Clinical net benefit for the positive endpoints was also assessed through decision curve analysis (Figure 4). Across threshold probabilities of 0.05–0.50, the radiomic model outperformed both strategies over 0.12–0.50 for cT status, 0.34–0.50 for cN status and 0.38–0.50 for clinical stage. At lower thresholds, the radiomic curves concurred with the treat-all strategy. At a probability of 0.40, the net benefit was 0.344 for the radiomic cT model versus 0.129 for the clinical baseline and 0.194 for treating all cases; 0.097 versus 0.065 and 0.032 for cN status; and 0.108 versus 0.129 and 0.032 for clinical stage. For cT status, the radiomic model exceeded the clinical baseline across most of the examined range. For clinical stage, the clinical baseline had a higher net benefit at most thresholds, and for cN status they alternated. This baseline is a minimal benchmark and does not represent contemporary clinical practice, which integrates complex clinical data, multiple imaging modalities, biopsy results and genetic testing.

### 3.6. Segmentation Reproducibility

In a 30-case second-reader subsample, the median Dice similarity coefficient between the original and the second reader’s tumor contours was 0.898 (mean: 0.854, range: 0.372–0.966). All 11 features retained by the three final models exceeded the threshold of 0.75, with ICCs ranging from 0.822 to 0.999; per-feature values with confidence intervals are presented in the Supplementary Table S4.

## 4. Discussion

The majority of radiomics research in BC is based on features derived from MR imaging. MRI provides much superior, three-dimensional visualization of the breast and tumor tissue. MRI-based radiomics predict complex outcomes, such as response to chemotherapy, presence of the triple-negative subtype, hormonal receptor status and Ki-67 expression, among others [5,17,18,19]. However, the modality is much less frequently used in clinical practice and rarely employed in screening or as an initial diagnostic method. By contrast, MMG is widely and easily available around the world, and it is most likely to be the first imaging performed when a suspicious breast mass is reported by a patient or identified by a physician during a physical examination. Therefore, reliably extracting useful clinical data from a widely available imaging modality would provide a much-needed solution for risk stratification and triage of patients, with the prioritization of further diagnosis in those with higher suspicion of advanced or metastatic disease, especially in the age of BC overdiagnosis and possible overtreatment [20,21]. The body of literature is more limited for MMG than for other modalities [22,23].

In this single-center study, we focused on the prediction of disease burden and PIK3CA status based on MMG-based radiomics. Among the studied endpoints, three—prediction of cN status, cT status and overall disease stage—showed statistically significant temporally validated discrimination after BH-FDR correction. The temporal split, fixed throughout the analyses, with the most recently diagnosed 30% of cases assigned to the test subgroup simulated prospective deployment.

Prediction of cN status using MMG-based radiomics was chosen as a primary endpoint for its clinical relevance to the disease risk stratification and choice between surgical intervention and neoadjuvant chemotherapy. Clinical status, based on ultrasound as per AJCC guidelines, was chosen instead of the surgical staging because the majority of the patients included in the study underwent neoadjuvant chemotherapy, often achieving postoperative partial or complete remission. Therefore, the surgical staging does not represent the initial nodal status at the point of MMG acquisition. The radiomic model achieved a temporally validated test set AUC of 0.726. Four leading features for the predictive power were related to tumor texture, derived from the MLO projection. They encode the intratumoral heterogeneity, and we hypothesize that this mammographic heterogeneity could reflect the underlying tumor biology associated with nodal involvement, potentially including stromal disorganization and microcalcification patterns previously linked to advanced disease [24,25]. Further work, optimally also encompassing radiopathomics, is needed to explore this theory. Until then, the connection of MMG-based radiomics to biological phenotype remains hypothetical. Previous research, including the work by Tan et al. and Yang et al., among others, confirms the ability of MMG-based radiomics to predict lymph node metastases [26,27]. A more detailed comparison of our work with previous studies on MMG-based radiomics prediction of nodal status in breast cancer is presented in the Supplementary Table S5. Although our test set AUC is lower than those of several of the previously reported MMG-based nodal status prediction models, this likely results from the more conservative temporal-split validation we applied. In both mentioned papers, random train-validation partitions of the cohort were used. They also assessed the pathological lymph node status (pN), different from our cN analysis. Additionally, in the Tan et al. paper, the prediction model was more complex, combining features extracted from both MRI and MMG.

Prediction of cT and the overall clinical stage by MMG-based radiomics is, by definition, partly explained by tumor size: tumor diameter, which can be measured directly on a mammogram, defines the cT category and is a major component of clinical stage. GLDZM large-distance high-gray-level emphasis, the dominant feature for the cT model, rewards tumor regions that have many high-intensity zones located deep inside the lesion. Therefore, this feature carries additional signal intensity and tumor texture components. The clinical stage prediction model retained a related set of intratumoral intensity and zone-distance descriptors (global intensity peak and GLDZM small-distance emphasis), which carry additional signal intensity and texture information. We hypothesize that this pattern is consistent with the biological phenotype of the disease and that some radiomic features track tumor aggressiveness. However, the pathological correlation was not analyzed in this study.

Two of the endpoints—the prediction of PIK3CA mutation and radiologist-assigned BI-RADS group—failed, with large CV-test AUC differences typical of overfitting and low EPV. Regarding PIK3CA status prediction, a genotype–phenotype relationship is possibly too subtle for mammographic resolution combined with reported low EPV. Nevertheless, the negative results are retained deliberately, not to overestimate the potential yield of MMG-based radiomics. Interestingly, recent work by Yao et al. focused on the prediction of PIK3CA mutation in BC and found positive results (AUC: 0.718) for the prediction of this genetic alteration and even stronger results for ultrasound-based radiomics and a combined model [28]. This contradicts our findings and could be either a result of a larger sample (186 patients) or a different statistical methodology. Further development of PIK3CA mutation presence prediction is key for earlier qualification for treatment with alpelisib in patients with advanced BC [29]. Nevertheless, the majority of the research on MMG-based radiomics still focuses on fundamental cancer risk stratification [30]. BI-RADS assessment reflects the radiologist’s judgment, which includes tumor margin, calcifications and surrounding tissue that lie outside the segmented region. The negative result possibly indicates a mismatch between endpoint and feature space.

We acknowledge the limitations of our study. First, the sample size is small given the number of variables produced by the radiomics software; EPV fell below the TRIPOD-recommended threshold, and the study was not powered to detect between-model AUC differences of the magnitude observed. With a test set of only 31 cases, the confidence intervals were expected to be wide, reducing the clinical applicability of the results. Feature selection was unstable for the cN and the overall clinical stage models, so the specific features reported are representative rather than definitive.

The study is single-center, with no external validation cohort. A further limitation is the suboptimal calibration of two of the positive endpoints: cN prediction, with a slope of 3.762 suggesting too conservative predictions, and cT prediction, with a slope of 0.444, typical of overfitting. Calibration plots are available in the Supplementary Figure S2. A recalibration on a larger, external cohort would benefit the clinical applicability of the results. The cohort included mammograms acquired on two systems (Siemens and Hologic). This heterogeneity arguably enhances the generalizability of our findings beyond a single-scanner setting. As a sensitivity analysis, we compared each model-selected feature between vendors in the subset of cases. None differed significantly (in all cases, Mann–Whitney *p* > 0.05). However, we emphasize that this is a sensitivity analysis, and not a substitute for formal radiomic harmonization (e.g., ComBat). The entire comparison is available in the Supplementary Table S6. Our IBSI-compliant pipeline applies standardized preprocessing and discretization to all images to partially mitigate this issue. Regarding the clinical baseline comparison, tumor size was taken from the pathology report and would therefore not have been available at the time of mammographic reporting, which is the intended moment of prediction. A mammographic tumor diameter was not recorded in a standardized form across the cohort, so pathological size was used. On the other hand, this choice is conservative, as pathological size makes the baseline stronger than any model available at the mammographic timepoint. Regarding intra-observer reproducibility assessment, we did not accommodate the washout period, and we did not quantify the test–retest variability of the same reader. The presented models remain hypothesis-generating until they are externally validated in multicenter cohorts.

We treat the pre-specified pipeline and the blinded, leakage-proof temporal test set as significant methodological strengths. IBSI-compliant feature extraction, multiple-testing correction across endpoints and retention of negative endpoints also strengthen the results. A larger, externally validated study is required to confirm our findings, in particular regarding the cN status prediction, and to properly compare them to the clinical baseline.

Before any clinical use can be considered, several important steps need to be taken. The first is external, multicenter validation. The validation should span multiple MMG vendors, acquisition protocols and readers for better generalizability of the results. Secondly, a prospective study would be required at the decision point the models are intended to inform. What is more, use of MMG-radiomics-based prediction software would require adherence to European Union and United States medical-device regulations, requiring documented risk management, clinical evaluation and surveillance. The larger question of how artificial intelligence (AI) tools in oncology can move from promising but experimental and retrospective performance to accountable clinical deployment is gaining traction [31]. Recent work on AI-assisted MMG interpretation illustrates both the potential of such tools and their dependence on the population in which they are deployed [32].

## 5. Conclusions

Our study provides preliminary evidence that radiomic data extracted from mammography may discriminate between cN0 and cN-positive disease and that tumor texture and intensity features track clinical tumor stage and overall disease burden in BC. As these associations were demonstrated in a single, modest cohort and did not significantly exceed a clinical baseline, they should not be interpreted as a clinically validated prediction model at this moment. Our study found no correlation between radiomic features and *PIK3CA* mutational status in BC patients and showed no concordance between radiomic features and radiologist-assessed BI-RADS group. Further—larger and externally validated—studies are needed to establish clinical utility and possibly examine other domains that can be predicted by MMG-derived radiomics.

## Figures and Tables

**Table 1:** Baseline characteristics of the study cohort (n = 102 cases from 100 patients).

Variable	Overall (n = 102)	Training (n = 71)	Test (n = 31)	*p* (Training vs. Test)
Age, years	63 [49–70]; 60.5 ± 13.4; 35–92	63 [51–70]; 60.6 ± 12.8	61 [49–73]; 60.2 ± 14.9	0.847
Age at diagnosis, years	59 [47–67]; 57.4 ± 13.7; 25–91	59 [48–65]; 56.7 ± 13.3	59 [48–72]; 58.8 ± 14.7	0.668
Tumor size from pathology report, mm (n = 73)	27 [20–35]; 33.0 ± 27.3; 5–200	29 [20–35]; 30.7 ± 19.1	27 [19–40]; 37.8 ± 39.1	0.746
Ki67, % (n = 97)	20 [10–60]; 32.3 ± 29.1	20 [6–38]; 26.0 ± 25.1	40 [15–70]; 45.8 ± 32.7	0.006
ER-positive (ER1-3), n (%)	85 (83.3)	61 (85.9)	24 (77.4)	0.224
ER-negative (ER0), n (%)	15 (14.7)	8 (11.3)	7 (22.6)	
PR-positive (PR1-3), n (%)	76 (74.5)	51 (71.8)	25 (80.6)	0.614
PR-negative (PR0), n (%)	24 (23.5)	18 (25.4)	6 (19.4)	
HER2-positive, n (%)	11 (10.8)	7 (9.9)	4 (12.9)	0.733
HER2-negative, n (%)	90 (88.2)	63 (88.7)	27 (87.1)	
Luminal B, n (%)	49 (48.0)	34 (47.9)	15 (48.4)	0.715
Luminal A, n (%)	29 (28.4)	22 (31.0)	7 (22.6)	
Luminal B HER2-positive, n (%)	7 (6.9)	4 (5.6)	3 (9.7)	
Triple-negative, n (%)	10 (9.8)	5 (7.0)	5 (16.1)	
HER2-enriched, n (%)	4 (3.9)	3 (4.2)	1 (3.2)	
NOS histology, n (%)	83 (81.4)	54 (76.1)	29 (93.5)	0.107
Lobular histology, n (%)	14 (13.7)	13 (18.3)	1 (3.2)	
Grade G1, n (%)	37 (36.3)	29 (40.8)	8 (25.8)	**0.034**
Grade G2, n (%)	48 (47.1)	35 (49.3)	13 (41.9)	
Grade G3, n (%)	16 (15.7)	7 (9.9)	9 (29.0)	
BI-RADS 4, n (%)	31 (30.4)	21 (29.6)	10 (32.3)	0.868
BI-RADS 5, n (%)	59 (57.8)	42 (59.2)	17 (54.8)	
BI-RADS 6, n (%)	5 (4.9)	4 (5.6)	1 (3.2)	
cT1, n (%)	13 (12.7)	8 (11.3)	5 (16.1)	0.352
cT2, n (%)	47 (46.1)	37 (52.1)	10 (32.3)	
cT3, n (%)	23 (22.5)	15 (21.1)	8 (25.8)	
cT4, n (%)	18 (17.6)	10 (14.1)	8 (25.8)	
cN0, n (%)	52 (51.0)	34 (47.9)	18 (58.1)	0.289
cN1, n (%)	41 (40.2)	32 (45.1)	9 (29.0)	
cN2/cN3, n (%)	9 (8.8)	5 (7.0)	4 (12.9)	
Stage I, n (%)	10 (9.8)	5 (7.0)	5 (16.1)	0.045
Stage II, n (%)	49 (48.0)	36 (50.7)	13 (41.9)	
Stage III, n (%)	27 (26.5)	15 (21.1)	12 (38.7)	
Stage IV, n (%)	15 (14.7)	14 (19.7)	1 (3.2)	
PIK3CA mutated, n (%)	42 (41.2)	31 (43.7)	11 (35.5)	0.511
Local recurrence, n (%)	9 (8.8)	5 (7.0)	4 (12.9)	0.446
Distant metastasis, n (%)	49 (48.0)	41 (57.7)	8 (25.8)	**0.005**
Follow-up time, OS, months (n = 101)	52 [40–65]	59 [49–78]	46 [28–50]	**<0.001**

Continuous variables: median [interquartile range, IQR]; mean ± standard deviation (SD); range. Categorical variables: n (%). Tumor size (from pathology report) missing in 29 cases (median-imputed whenever used in analyses). The *p*-values compare the training and test subsets once per variable: Mann–Whitney U test for continuous variables, Fisher’s exact test for two-level categorical variables, and a Monte Carlo permutation chi-square (10,000 permutations) for variables with more than two levels, all of which had an expected cell count below five. Cases with a missing value for a given variable were excluded from that variable’s test. Categories not tabulated were omitted. Three variables differed at *p* < 0.05 after Benjamini–Hochberg correction. The shorter follow-up and lower observed metastasis rate in the test subset resulted from the temporal split. The higher Ki67 in the test subset could correspond to changes in diagnostic practice over time. Abbreviations: ER, estrogen receptor; PR, progesterone receptor; HER2, human epidermal growth factor receptor 2; NOS, not otherwise specified; G, grade; cN, clinical nodal status; cT, clinical tumor stage; BI-RADS, Breast Imaging Reporting and Data System; OS, overall survival.

**Table 2:** Primary results of the analysis.

Endpoint	n	Prevalence	Representation	Model	CV AUC	Test AUC (95% CI)	Permutation *p*-Value	B-H q-Value
cN status (0 vs. 1+)	102	49.0%	Tumor texture, MLO	L1 + L2	0.684	**0.726 (0.501–0.886)**	**0.010**	**0.017**
Clinical stage (I–II vs. III–IV)	101	41.6%	All tumor features, MLO	L1 + elastic net	0.763	**0.778 (0.572–0.917)**	**0.003**	**0.010**
cT (1–2 vs. 3–4)	102	40.2%	Tumor texture (combined projections)	ANOVA + elastic net	0.824	**0.792 (0.575–0.929)**	**0.004**	**0.010**
*PIK3CA *mutation status (positive vs. negative)	100	42.0%	All tumor features	L1 + L2	0.721	0.432 (0.227–0.657)	0.733	0.917
BI-RADS group (4 vs. 5)	90	65.6%	Tumor + asymmetry	L1 + elastic net	0.816	0.300 (0.131–0.537)	0.962	0.962

CV: 5 × 3 repeated stratified k-fold cross-validation on training set. Test AUC: temporally held-out 30% subset. 95% CI: bias-corrected bootstrap (2000 resamples). Permutation *p*-value: 1000-permutation test on the test set. B-H q-value: Benjamini–Hochberg false discovery rate adjustment (α = 0.05). Abbreviations: cN, clinical nodal status; cT, clinical tumor stage; BI-RADS, Breast Imaging Reporting and Data System; CV, cross-validation; AUC, area under the receiver operating characteristic curve; CI, confidence interval; CC, craniocaudal; MLO, mediolateral oblique.

**Table 3:** Bootstrap internal validation of the positive endpoints (‘optimism correction’).

Endpoint	Apparent AUC	Optimism	‘Optimism-Corrected’ AUC	Temporal-Test AUC
cN status (0 vs. 1+)	0.691	0.034	0.656	0.726
cT status (1–2 vs. 3–4)	0.830	0.053	0.777	0.792
Clinical stage (I–II vs. III–IV)	0.761	0.030	0.732	0.778

Abbreviations: cN, clinical nodal status; cT, clinical tumor stage; AUC, area under the receiver operating characteristic curve; CI, confidence interval.

**Table 4:** Selected features for the positive endpoints after model refit on all available cases.

Endpoint	Feature	Coefficient	Folds Selected (out of 15)
cN status	cm_info_corr1_2D_avg (Tumor MLO)	−0.05	4 (27%)
	dzm_gl_var_2D (Tumor MLO)	+0.05	<2 (<13%)
	dzm_zd_entr_2D (Tumor MLO)	+0.14	10 (67%)
	ngl_ldlge_2D (Tumor MLO)	−0.10	3 (20%)
Clinical stage	dzm_sde_2D (Tumor MLO)	−0.19	<2 (<13%)
	dzm_sdlge_2D (Tumor MLO)	0	<2 (<13%)
	loc_peak_glob (Tumor MLO)	+0.36	<2 (<13%)
cT status	dzm_ldhge_2D (Tumor CC)	+0.90	15 (100%)
	dzm_zd_var_2D (Tumor CC)	0	7 (47%)
	ngt_complexity (Tumor CC)	+0.60	8 (53%)
	rlm_rlnu_2D_avg (Tumor CC)	−0.05	2 (13%)

Coefficient of 0 implies that L1 eliminated the feature in the final model despite its inclusion in the feature subset. The stability column shows selection frequency across the 15 cross-validation folds (5 × 3 repeats). Abbreviations: cN, clinical nodal status; cT, clinical tumor stage; CC, craniocaudal; MLO, mediolateral oblique. Definitions of the selected radiomic features are available in the Supplementary Table S1.

**Table 5:** Calibration metrics with 95% confidence intervals.

Endpoint	Brier Score (95% CI)	Calibration Intercept (95% CI)	Calibration Slope (95% CI)
cN status	0.229 (0.209 to 0.249)	−0.431 (−1.636 to +0.359)	+3.762 (0.951 to 11.174)
Clinical stage	0.211 (0.180 to 0.244)	−0.409 (−1.526 to +0.492)	+2.542 (0.827 to 5.853)
cT status	0.202 (0.116 to 0.302)	+0.052 (−0.688 to +2.844)	+0.444 (0.007 to 3.563)
PIK3CA status	0.337 (0.234 to 0.438)	−0.657 (−1.686 to +0.177)	−0.063 (−0.570 to +0.382)
BI-RADS group	0.342 (0.267 to 0.424)	+0.881 (0.037 to 2.210)	−0.828 (−2.206 to −0.049)

Abbreviations: cN, clinical nodal status; cT, clinical tumor stage; BI-RADS, Breast Imaging Reporting and Data System; CI, confidence interval; AUC, area under the receiver operating characteristic curve.

**Table 6:** Performance of the radiomic models in comparison with the clinical baseline (age ± BI-RADS group ± tumor size).

Endpoint	Clinical AUC	Radiomic AUC	DeLong p (Radiomic vs. Clinical)
cN status	0.697	0.726	0.820
Clinical stage	0.726	0.778	0.687
cT status	0.537 *	0.792	0.108
*PIK3CA *status	0.605	0.432	0.285
BI-RADS group	0.592 *	0.300	0.034

* For cT prediction, a clinical model included only age at diagnosis and BI-RADS group. For BI-RADS prediction, the clinical model included only age at diagnosis and tumor size. Abbreviations: cN, clinical nodal status; cT, clinical tumor stage; BI-RADS, Breast Imaging Reporting and Data System; CV, cross-validation; AUC, area under the receiver operating characteristic curve.

## Data Availability

The MMG datasets cannot be publicly shared as per EU data protection regulations. Anonymized, extracted radiomic data is available on request.

## Supplementary Materials

The following supporting information can be downloaded at https://www.mdpi.com/article/10.3390/cancers18162566/s1: Table S1. Extracted radiomic features dictionary. Table S2. Data missingness report. Table S3. PROBAST-AI-style risk-of-bias self-assessment. Table S4. Inter-observer reproducibility of the ra-diomic features retained by the three final models. Table S5. Comparison with previous mammog-raphy-based radiomics papers. Table S6. Comparison of model-selected radiomic features between mammography. Figure S1. Precision-recall curves for the positive endpoints. Figure S2. Calibration curves for the positive endpoints.
