# Supplementary material for: Mammography-Based Radiomics for Prediction of Nodal Status and Disease Burden in Breast Cancer: A Temporally Validated Study
Source: Cancers (Basel). 2026 Aug 10;18(16):2566. doi: 10.3390/cancers18162566 (PMC13510888; doi:10.3390/cancers18162566)
Supplement: Supplementary file 1 [file cancers-18-02566-s001.zip › cancers-4434346-supplementary.pdf]

Supplementary Material

Table S1. Extracted radiomic features dictionary

Abbreviation	IBSI feature name
**First-order statistical (stat_*)**	
stat_mean	Mean intensity
stat_var	Intensity variance
stat_skew	Intensity skewness
stat_kurt	Intensity (excess) kurtosis
stat_median	Median intensity
stat_min	Minimum intensity
stat_max	Maximum intensity
stat_p10	10th intensity percentile
stat_p90	90th intensity percentile
stat_iqr	Interquartile range
stat_range	Intensity range
stat_mad	Mean absolute deviation
stat_rmad	Robust mean absolute deviation
stat_medad	Median absolute deviation
stat_cov	Coefficient of variation
stat_qcod	Quartile coefficient of dispersion
stat_energy	Energy
stat_rms	Root mean square intensity
**Intensity histogram (ih_*)**	
ih_mean	Mean discretised intensity
ih_var	Discretised intensity variance
ih_skew	Discretised intensity skewness
ih_kurt	Discretised intensity (excess) kurtosis
ih_median	Median discretised intensity
ih_min	Minimum discretised intensity
ih_max	Maximum discretised intensity
ih_mode	Mode of discretised intensity histogram
ih_p10	10th discretised intensity percentile
ih_p90	90th discretised intensity percentile
ih_iqr	Discretised intensity interquartile range
ih_range	Discretised intensity range
ih_mad	Intensity histogram mean absolute deviation
ih_rmad	Intensity histogram robust mean absolute deviation
ih_medad	Intensity histogram median absolute deviation
ih_cov	Intensity histogram coefficient of variation
ih_qcod	Intensity histogram quartile coefficient of dispersion
ih_entropy	Discretised intensity entropy
ih_uniformity	Discretised intensity uniformity
ih_max_grad	Maximum histogram gradient
ih_max_grad_g	Maximum histogram gradient grey level
ih_min_grad	Minimum histogram gradient
ih_min_grad_g	Minimum histogram gradient grey level
**Local intensity (loc_*)**	
loc_peak_loc	Local intensity peak

loc_peak_glob	Global intensity peak
**Grey Level Co-occurrence Matrix — GLCM (cm_*_2D_avg)**	
cm_joint_max_2D_avg	Joint maximum
cm_joint_avg_2D_avg	Joint average
cm_joint_var_2D_avg	Joint variance
cm_joint_entr_2D_avg	Joint entropy
cm_diff_avg_2D_avg	Difference average
cm_diff_var_2D_avg	Difference variance
cm_diff_entr_2D_avg	Difference entropy
cm_sum_avg_2D_avg	Sum average
cm_sum_var_2D_avg	Sum variance
cm_sum_entr_2D_avg	Sum entropy
cm_energy_2D_avg	Angular second moment (energy)
cm_contrast_2D_avg	Contrast
cm_dissimilarity_2D_avg	Dissimilarity
cm_inv_diff_2D_avg	Inverse difference
cm_inv_diff_norm_2D_avg	Normalised inverse difference
cm_inv_diff_mom_2D_avg	Inverse difference moment
cm_inv_diff_mom_norm_2D_avg	Normalised inverse difference moment
cm_inv_var_2D_avg	Inverse variance
cm_corr_2D_avg	Correlation
cm_auto_corr_2D_avg	Auto-correlation
cm_clust_tend_2D_avg	Cluster tendency
cm_clust_shade_2D_avg	Cluster shade
cm_clust_prom_2D_avg	Cluster prominence
cm_info_corr1_2D_avg	Information correlation 1
cm_info_corr2_2D_avg	Information correlation 2
**Grey Level Run Length Matrix — GLRLM (rlm_*_2D_avg)**	
rlm_sre_2D_avg	Short runs emphasis
rlm_lre_2D_avg	Long runs emphasis
rlm_lgre_2D_avg	Low grey level run emphasis
rlm_hgre_2D_avg	High grey level run emphasis
rlm_srlge_2D_avg	Short run low grey level emphasis
rlm_srhge_2D_avg	Short run high grey level emphasis
rlm_lrlge_2D_avg	Long run low grey level emphasis
rlm_lrhge_2D_avg	Long run high grey level emphasis
rlm_glnu_2D_avg	Grey level non-uniformity
rlm_glnu_norm_2D_avg	Normalised grey level non-uniformity
rlm_rlnu_2D_avg	Run length non-uniformity
rlm_rlnu_norm_2D_avg	Normalised run length non-uniformity
rlm_r_perc_2D_avg	Run percentage
rlm_gl_var_2D_avg	Grey level variance
rlm_rl_var_2D_avg	Run length variance
rlm_rl_entr_2D_avg	Run entropy
**Grey Level Size Zone Matrix — GLSZM (szm_*_2D)**	
szm_sze_2D	Small zone emphasis
szm_lze_2D	Large zone emphasis
szm_lgze_2D	Low grey level zone emphasis
szm_hgze_2D	High grey level zone emphasis
szm_szlge_2D	Small zone low grey level emphasis

szm_szhge_2D	Small zone high grey level emphasis
szm_lzlge_2D	Large zone low grey level emphasis
szm_lzhge_2D	Large zone high grey level emphasis
szm_glnu_2D	Grey level non-uniformity
szm_glnu_norm_2D	Normalised grey level non-uniformity
szm_zsnu_2D	Zone size non-uniformity
szm_zsnu_norm_2D	Normalised zone size non-uniformity
szm_z_perc_2D	Zone percentage
szm_gl_var_2D	Grey level variance
szm_zs_var_2D	Zone size variance
szm_zs_entr_2D	Zone size entropy
**Grey Level Distance Zone Matrix — GLDZM (dzm_*_2D)**	
dzm_sde_2D	Small distance emphasis
dzm_lde_2D	Large distance emphasis
dzm_lgze_2D	Low grey level zone emphasis
dzm_hgze_2D	High grey level zone emphasis
dzm_sdlge_2D	Small distance low grey level emphasis
dzm_sdhge_2D	Small distance high grey level emphasis
dzm_ldlge_2D	Large distance low grey level emphasis
dzm_ldhge_2D	Large distance high grey level emphasis
dzm_glnu_2D	Grey level non-uniformity
dzm_glnu_norm_2D	Normalised grey level non-uniformity
dzm_zdnu_2D	Zone distance non-uniformity
dzm_zdnu_norm_2D	Normalised zone distance non-uniformity
dzm_z_perc_2D	Zone percentage
dzm_gl_var_2D	Grey level variance
dzm_zd_var_2D	Zone distance variance
dzm_zd_entr_2D	Zone distance entropy
**Neighbourhood Grey Tone Difference Matrix — NGTDM (ngt_*)**	
ngt_coarseness	Coarseness
ngt_contrast	Contrast
ngt_busyness	Busyness
ngt_complexity	Complexity
ngt_strength	Strength
**Neighbouring Grey Level Dependence Matrix — NGLDM (ngl_*_2D)**	
ngl_lde_2D	Low dependence emphasis
ngl_hde_2D	High dependence emphasis
ngl_lgce_2D	Low grey level count emphasis
ngl_hgce_2D	High grey level count emphasis
ngl_ldlge_2D	Low dependence low grey level emphasis
ngl_ldhge_2D	Low dependence high grey level emphasis
ngl_hdlge_2D	High dependence low grey level emphasis
ngl_hdhge_2D	High dependence high grey level emphasis
ngl_glnu_2D	Grey level non-uniformity
ngl_glnu_norm_2D	Normalised grey level non-uniformity
ngl_dcnu_2D	Dependence count non-uniformity
ngl_dcnu_norm_2D	Normalised dependence count non-uniformity
ngl_dc_perc_2D	Dependence count percentage
ngl_gl_var_2D	Grey level variance
ngl_dc_var_2D	Dependence count variance

ngl_dc_entr_2D	Dependence count entropy
ngl_dc_energy_2D	Dependence count energy

Abbreviations: IBSI, Imaging Biomarker Standardization Initiative.

Table S2. Data missingness report.

	**Variable**	**n complete**	**n missing**	**% missing**	**n analyzed**	**Handling**
**A. Outcome labels**	cN status (0 vs 1+)	102	0	0.0	102	Complete-case exclusion (outcomes never imputed)
	cT status (1-2 vs 3-4)	102	0	0.0	102	Complete-case exclusion (outcomes never imputed)
	Clinical stage (I-II vs III-IV)	101	1	1.0	101	Complete-case exclusion (outcomes never imputed)
	PIK3CA status (wild-type vs mutated)	100	2	2.0	100	Complete-case exclusion (outcomes never imputed)
	BI-RADS category (4 vs 5)	90	12	11.8	90	Complete-case exclusion (outcomes never imputed)
**B. Clinical variables**	Age at diagnosis (years) [Clinical	102	0	0.0		Median imputation within CV pipeline

	baseline model + Table 1]					
	Age at mammography (years) [Table 1 (descriptive)]	102	0	0.0		None required (descriptive only)
	BI-RADS category [Clinical baseline model + Table 1]	95	7	6.9		Median imputation within CV pipeline
	Ki67 (%) [Table 1 (descriptive)]	97	5	4.9		None required (descriptive only)
	Index date (temporal split variable)	102	0	0.0		Complete-case exclusion
**C. Radiomic features**	Tumor ROI, CC view (138 features)	94	8	7.8		Median imputation within CV pipeline
	Tumor ROI, MLO view (138 features)	94	8	7.8		Median imputation within CV pipeline
	Whole breast, ipsilateral, CC view (138 features)	96	6	5.9		Median imputation within CV pipeline
	Whole breast, ipsilateral, MLO view (138 features)	96	6	5.9		Median imputation within CV pipeline
	Whole breast, contralateral, CC view (138 features)	84	18	17.6		Median imputation within CV pipeline
	Whole breast, contralateral,	86	16	15.7		Median imputation

	MLO view (138 features)					within CV pipeline
	Ipsilateral-contralateral difference, CC view (138 features)	84	18	17.6		Median imputation within CV pipeline
	Ipsilateral-contralateral difference, MLO view (138 features)	86	16	15.7		Median imputation within CV pipeline
	Image-availability count variables (8 features)	102	0	0.0		Complete by constructio n
**D. Imputed cases in final models**	cN status (0 vs 1+) — winning representation Tumor texture MLO (95 features)	94	8	7.8	8 train / 0 test	All model inputs median-imputed for these cases
	cT status (1-2 vs 3-4) — winning representation Tumor texture (190 features)	100	2	2.0	2 train / 0 test	All model inputs median-imputed for these cases
	Clinical stage (I-II vs III-IV) — winning representation Tumor all MLO (138 features)	93	8	7.9	8 train / 0 test	All model inputs median-imputed for these cases

Table S3. PROBAST-AI-style risk-of-bias self-assessment.

Domain	Risk of bias	Justification

**Participants**	**Low**	Consecutive histopathologically-confirmed breast cancer cases with available pre-treatment digital mammography from a single institution. Explicit inclusion/exclusion with a CONSORT-style flow diagram.
**Predictors**	**High**	IBSI-compliant radiomic features extracted with a standardized, pre-registered pipeline. Tumor segmentation guided by the written radiologist report. Single-reader segmentation with no inter-/intra-observer reproducibility analysis. Feature extraction was blind to endpoint.
**Outcome**	**Low**	Endpoints (cN, cT, stage, PIK3CA, BI-RADS) determined from clinical/pathological records independently of radiomic features. BI-RADS 6 excluded from analyses. BI-RADS category removed from the clinical baseline for the BI-RADS endpoint to prevent leakage.
**Analysis**	**High**	EPV below the conventional threshold of 10 for several endpoints; feature-selection instability for cN and stage; wide confidence intervals resulting from a 31-case test set, no external validation.<br>Mitigation methods: temporal held-out test set evaluated only once, model selection by CV AUC only, BH-FDR control, bootstrap optimism correction, calibration and decision-curve analyses reported.<br>Despite mitigation methods, the sample size leads to high bias risk.

Figure S1. Precision-recall curves for the positive endpoints.

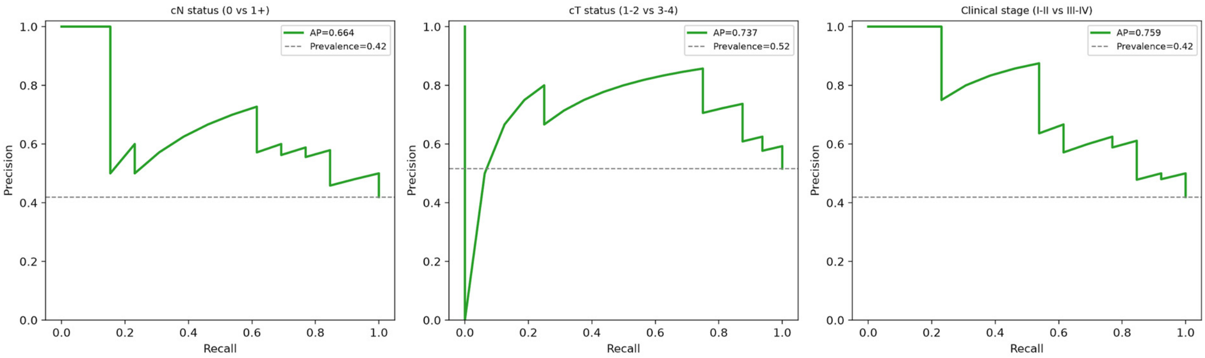

Table S4. Inter-observer reproducibility of the radiomic features retained by the three final models.

Endpoint	Projection	Feature	Feature class	ICC (2,1) (95% CI)
cN status (0 vs 1+)	MLO	cm_info_corr1_2D_avg	GLCM	0.928 (0.822-0.968)
cN status (0 vs 1+)	MLO	dzm_gl_var_2D	GLDZM	0.874 (0.685-0.968)
cN status (0 vs 1+)	MLO	dzm_zd_entr_2D	GLDZM	0.933 (0.844-0.976)
cN status (0 vs 1+)	MLO	ngl_ldlge_2D	NGLDM	0.822 (0.640-0.934)
cT status (1-2 vs 3-4)	CC	dzm_ldhge_2D	GLDZM	0.914 (0.861-0.959)
cT status (1-2 vs 3-4)	CC	dzm_zd_var_2D	GLDZM	0.855 (0.693-0.970)
cT status (1-2 vs 3-4)	CC	ngt_complexity	NGTDM	0.903 (0.821-0.933)
cT status (1-2 vs 3-4)	CC	rlm_rlnu_2D_avg	GLRLM	0.946 (0.845-0.982)
Clinical stage (I-II vs III-IV)	MLO	dzm_sde_2D	GLDZM	0.872 (0.682-0.958)
Clinical stage (I-II vs III-IV)	MLO	dzm_sdlge_2D	GLDZM	0.917 (0.719-0.964)
Clinical stage (I-II vs III-IV)	MLO	loc_peak_glob	Local intensity	0.999 (0.998-1.000)

Abbreviations: ICC, intraclass correlation coefficient; CI, confidence interval; MLO, mediolateral oblique; CC craniocaudal.

30 tumor-side mammograms (15 CC, 15 MLO), one per patient, were randomly sampled and segmented by a second reader who was blinded to the original contour, then features were reextracted with Z-Rad, utilizing the initial settings described in the Methods section.
ICC (2,1) is a two-way random-effect model for the absolute agreement of single measurements.
Median Dice similarity between the original and the second-reader tumor ROIs was 0.898 (mean 0.854, range 0.372-0.966).
All 11 features retained by the three final models exceeded the prespecified ICC threshold of 0.75, the lowest being 0.822.

Table S5. Comparison with previous mammography-based radiomics papers.

Study	n	Centers	Validation strategy	Nodal endpoint	Reported AUC (validation)
Yang et al. 2019	147	1	Random split (3:1), internal	Pathological ALN metastasis	0.875 (signature)
Tan et al. 2020	216	1	Random split (2:1), internal	Pathological ALN metastasis	0.863 (nomogram)
Wang et al. 2024	485	2	Independent external	ALN metastasis (MRI + MMG)	0.892 (external, multimodal)
**Present study**	**102**	**1**	**Fixed temporal split (newest 30% held out)**	**Imaging-defined clinical nodal status (cN, pre-treatment)**	**0.726**

Abbreviations: AUC, area under the curve; ALN, axillary lymph nodes; MRI, magnetic resonance imaging; MMG, mammography; cN, clinical nodal status

Figure S2. Calibration curves for the positive endpoints.

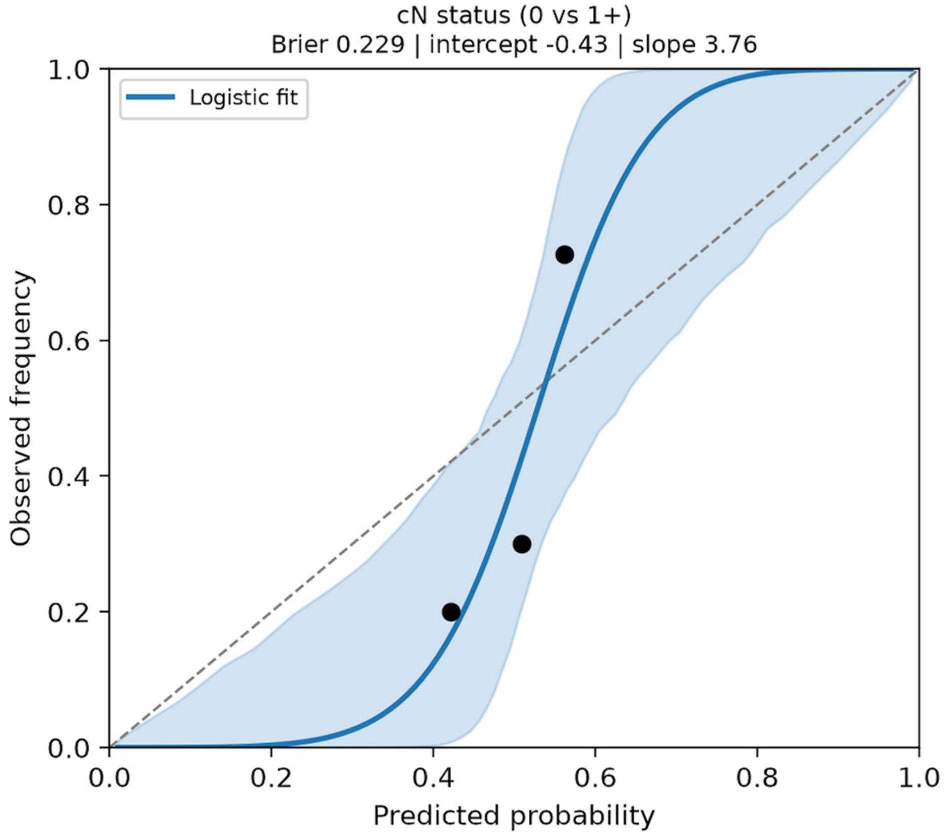

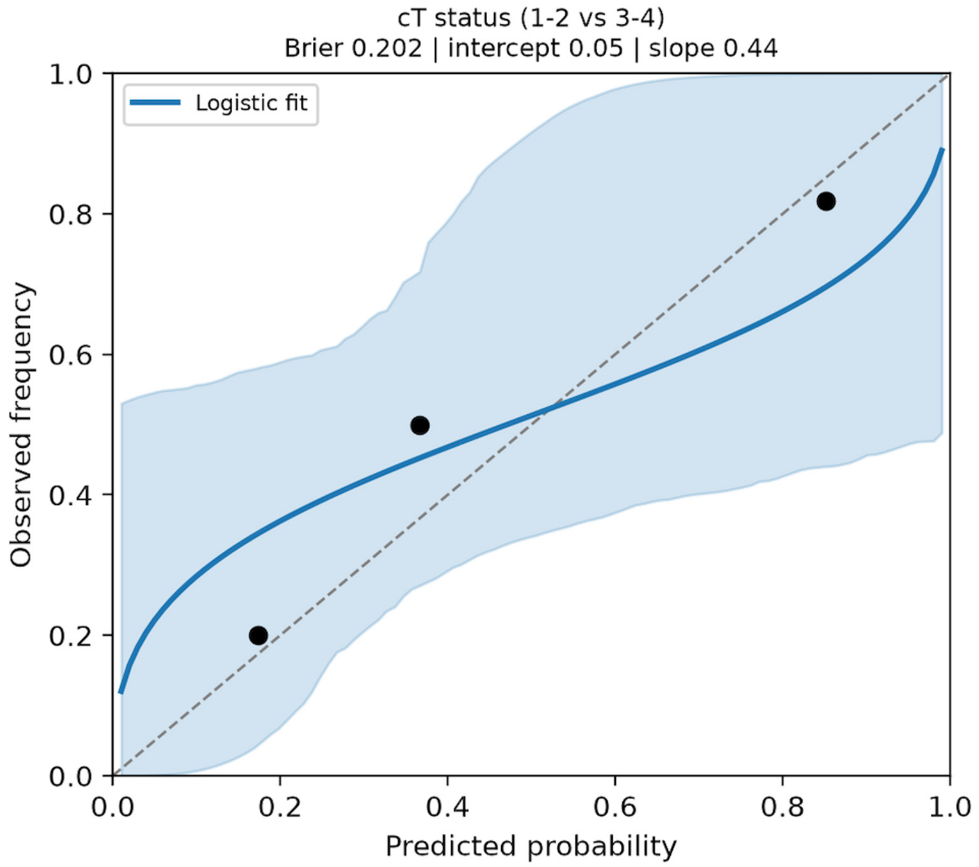
cT status (1-2 vs 3-4)
Brier 0.202 | intercept 0.05 | slope 0.44
Logistic fit
Observed frequency
Predicted probability
0.0
0.2
0.4
0.6
0.8
1.0

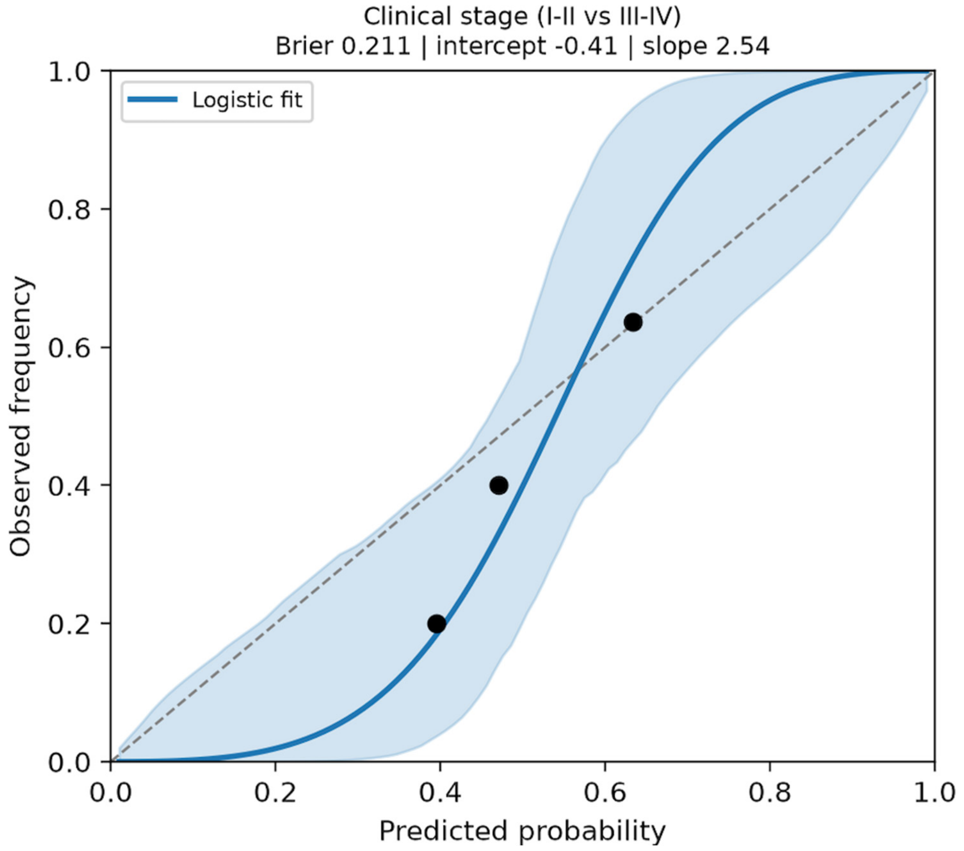
Clinical stage (I-II vs III-IV)
Brier 0.211 | intercept -0.41 | slope 2.54
Logistic fit
Observed frequency
Predicted probability
0.0
0.2
0.4
0.6
0.8
1.0

Table S6. Comparison of model-selected radiomic features between mammography vendors (Siemens versus Hologic).

Endpoint	Feature	n_siemens	n_hologic	median_siemens	median_hologic	Mann-Whitney p-value
cN status	cm_info_corr1_2D_avg	33	26	-0.475	-0.478	0.7893
cN status	dzm_gl_var_2D	33	26	298.45	268.982	0.5773
cN status	dzm_zd_entr_2D	33	26	12.469	12.278	0.9696
cN status	ngl_ldlge_2D	33	26	0	0	0.0976
cT status	dzm_ldhge_2D	31	26	11861902.99	12873369.49	0.6711
cT status	dzm_zd_var_2D	31	26	1018.101	960.71	0.8163
cT status	ngt_complexity	31	26	172723724.5	261445201.5	0.5695
cT status	rlm_rlnu_2D_avg	31	26	41794.824	41707.958	0.8288
Stage	dzm_sde_2D	33	26	0.034	0.035	0.7198
Stage	dzm_sdlge_2D	33	26	0	0	0.4777
Stage	loc_peak_glob	33	26	2966.998	3138.528	0.3718

Abbreviations: cN, clinical nodal status; cT, clinical tumor status.
